# Women’s autonomy and discrimination relevant to cervical cancer screening

**DOI:** 10.3389/frph.2026.1837034

**Published:** 2026-08-14

**Authors:** Adja Maguette Diaw, Fatoumata Binetou Diongue, Ibrahima Ndiaye, Omar Gassama, Ndeye Mbombe Dieng, Saria Awadalla, Fatou Bintou Seck, Ndèye Marème Sougou, Sarah Abboud, Assane Sall, Hilaire Sarr, Aida Fall, Aliath Salami, Alexis Claire Klima, Aida Barro, Elle Martell, Ahmadou Babana El Alaoui, J Andrew Dykens, Caryn E. Peterson, Adama Faye

**Affiliations:** 1 Institute of Health and Development, Cheikh Anta Diop University, Dakar, Senegal; 2 Office of Planning, Monitoring, Evaluation, and Research, Division of Maternal and Child Health, Ministry of Health and Social Action, Dakar, Senegal; 3 Obstetrics and Gynecology, Aristide Le Dantec Teaching Hospitals, Cheikh Anta Diop University, Dakar, Senegal; 4 Center for Training in Women’s Health, Cheikh Anta Diop University, Dakar, Senegal; 5 Office of Cancer Control, Division for the Control of Noncommunicable Diseases, Ministry of Health and Social Action, Dakar, Senegal; 6 Division of Epidemiology & Biostatistics, School of Public Health, University of Illinois Chicago, Chicago, IL, United States; 7 Department of Human Development Nursing Science, College of Nursing, University of Illinois Chicago, Chicago, IL, United States; 8 Department of Family and Community Medicine, University of Illinois Chicago, Chicago, IL, United States; 9 College of Applied Health Sciences, University of Illinois Chicago, Chicago, IL, United States; 10 Division of Epidemiology & Biostatistics at the School of Public Health, University of Illinois Chicago, Chicago, IL, United States; 11 College of Medicine, University of Illinois Chicago, Chicago, IL, United States; 12 Center for Global Health at the College of Medicine, University of Illinois Chicago, Chicago, IL, United States; 13 Cancer Center, University of Illinois Chicago, Chicago, IL, United States

**Keywords:** autonomy, cervical cancer, screening, Senegal, women

## Abstract

**Introduction:**

Cervical cancer remains the fourth most common cancer affecting women worldwide, with large geographical variations in incidence and mortality rates. The objective of this study is to determine whether women's autonomy is predictive of the use of cervical cancer screening services in Senegal.

**Method:**

This was a descriptive and analytical cross-sectional study of a sample of 312 women. Data were analyzed using R 4.2 software. Logistic regression was used to analyze associations between indicators of women's autonomy and cervical cancer screening practices.

**Results:**

Compared with women whose partners made the decision regarding cervical cancer screening, women who reported making the decision themselves (AOR: 10.07; 95% CI: 2.72, 37.33) or jointly with their partner (AOR: 5.34; 95% CI: 1.35, 21.21) were more likely to have undergone screening. Women who were aware of the existence of a cervical cancer screening test were also more likely to have been screened than those who were not aware of it (AOR: 7.67; 95% CI: 1.41, 41.61). In contrast, women residing in Dakar were more likely to have undergone cervical cancer screening than those residing in the Kédougou region (AOR: 0.40; 95% CI: 0.17, 0.96).

**Conclusion:**

This study shows that increased autonomy among women predicts greater use of cervical cancer screening services by women. Therefore, policy interventions should focus on empowering women to make autonomous decisions about their health.

## 1 Introduction

Cervical cancer is among the most common cancers affecting women worldwide, ranking fourth in frequency, with substantial regional variations in incidence and mortality (1). According to estimates from the World Health Organization, in the absence of additional measures, the annual number of new cervical cancer cases could increase from 570,000 to 700,000 between 2018 and 2030, while the number of deaths could rise from 311,000 to 400,000 (1). Furthermore, nearly 90% of cases and deaths reported in 2020 occurred in low- and middle-income countries. Comparative evidence from multiple countries has also shown that those with organized screening programs have experienced a gradual decline in both incidence and mortality, whereas many countries in sub-Saharan Africa have reported increasing trends (2). Therefore, the implementation of effective screening and early detection interventions could substantially reduce cervical cancer-related mortality as well as the associated health and social costs (3). However, the uptake of cervical cancer screening remains influenced by a range of individual, social, and cultural factors, including beliefs, stigma, educational level, socioeconomic status, gender roles, geographic characteristics, age, parity, marital status, and women's autonomy (4). In this context, women's autonomy can be understood as their ability to access information, exercise decision-making power, and act independently regarding their own health and that of their family (5).

This study aims to explore the relationship between women's empowerment and cervical cancer screening uptake in order to inform public health interventions in this area. The study was guided by Andersen and Newman's Behavioral Model of Health Service Utilization, which posits that the use of health services is influenced by predisposing factors, enabling factors, and perceived need for care. Within this framework, women's autonomy can be considered an enabling factor because it influences their ability to make decisions, access information, mobilize resources, and seek healthcare services. This model provides a useful framework for understanding how women's decision-making power may affect the uptake of cervical cancer screening services in Senegal (6). More specifically, this study seeks to determine whether women's autonomy is a predictive factor for the use of cervical cancer screening services in Senegal.

## 2 Study setting

Senegal, located at the western tip of Africa, is divided into 14 administrative regions and covers an area of 196,712 km^2^. Its population is estimated at 18,032,473, unevenly distributed across the country (7). This study was conducted in the regions of Dakar and Kédougou, which represent two contrasting socio-economic and health contexts (8). The Dakar region, which is predominantly urban, accounts for around 22% of the national population, with a very high density of 7,277 inhabitants per km^2^. Conversely, the Kédougou region, which is mainly rural, is the least populated in the country, with a density of only 15 inhabitants per km^2^.

Socioeconomically, there are significant disparities between urban and rural areas in the country. In rural areas, 53.6% of the population lives below the national poverty line, compared to 19.8% in urban areas. Furthermore, 75.4% of poor people live in rural areas (9). Access to education also reflects these inequalities: the gross enrollment rate is 18.2% in preschool, 81.0% in primary school, 50.6% in middle school, and 30.3% in secondary school, with rates consistently higher in urban areas (8).

The Senegalese health system is based on a network of 44 public health centers, unevenly distributed across the country. The Dakar region is home to the majority of the country's referral health facilities and employs 38.9% of the country's qualified health personnel. In contrast, the Kédougou region has only 1.4% of these human resources (10). Dakar comprises the health districts of Keur Massar, Diamniadio, and Yeumbeul, while Kédougou comprises the districts of Kédougou, Saraya, and Salemata. Each district has a health center located in the departmental capital, as well as several health posts located in the surrounding rural areas.

## 3 Methodology

This study is a cross-sectional descriptive and analytical analysis based on baseline data collected as part of a larger longitudinal study. The parent study includes a baseline survey followed by annual follow-up assessments. The present analysis uses only baseline data to examine factors associated with cervical cancer screening uptake among women in Senegal. In each health district (cluster), the referral health center and two randomly selected health posts were included (Table 1) (11). The study population consisted of women aged 25 to 69 residing in the districts of Keur Massar, Yeumbeul, Diamniadio, Kédougou, Salémata who were eligible for cervical cancer screening and living in a household within the study area.

**Table 1:** Survey areas and intervention application by stage.

Level	Dakar	Kédougou
District	Keur Massar	Saraya
Health center (HC)	Keur Massar HC	Saraya HC
Health post (HP)	Mme Fatou Ba HP	Dimboli HP
	Boune HP	Fongolimby HP
District	Yeumbeul	Salémata
Health center	Yeumbeul HC	Salémata HC
Health post	Yeumbeul Nord Maternity Hospital HP	Bembou HP
	Yeumbeul Diamalaye HP	Diakha Macky HP
District	Diamniadio	Kédougou
Center de santé	Diamniadio HC	Kédougou HC
Health post	Niangal HP	Darou Ningou HP
	Sébikotane HP	Darou Salam HP

### 3.1 Sampling procedure

We used the recommended sample size calculation formula for measuring changes in indicators for this study. The formula included adding a 10% non-response rate and a 10% loss to follow-up rate. The sample size included approximately 360 eligible women, spread across six districts. Recruitment utilized a two-stage cluster sampling methodology with a probability proportional to size and without stratification to select women and their partners to be surveyed. Stage one included the selection of census districts (CD), geographical areas with precise boundaries composed of households, as the primary unit associated with the health structure coverage areas. This included three CDs per health center and two CDs per health post. Stage two included a systematic random selection of households within the 42 sampled CDs. At health center sites, ten households were randomly selected in each CD, for a total of 30 women. In health posts, ten households were selected in one CD and five in another CD, for a total of 15 women (11).

### 3.2 Data collection

A total of three questionnaires were developed for the target population of our study. The first questionnaire was used to obtain the participants’ consent. The second questionnaire was used to collect demographic data, and the third focused on data on perceptions and knowledge, particularly intrapersonal barriers (knowledge/awareness, factual knowledge, personal attitudes, social attitudes), household barriers, financial considerations, structural/societal barriers (decision-making/autonomy), behavior, and experience with cervical cancer screening.

Field workers underwent three days of training covering all aspects of the survey and the use of tablets and the REDCap application for data collection. The training was followed by a one-day pre-test in a location other than the survey site. Data were collected through face-to-face interviews where questions were asked by the interviewers in the individual participant's local languages (French, Wolof, Malinké, Pulaar, Diahanké, etc.). Male interviewers were interviewed by men and women were also interviewed by female interviewers. The application used for data collection was developed with REDCap software. The data collected was sent to REDCap, which stored the information.

### 3.3 Description of variables

Cervical cancer screening was the dependent variable in the study. To obtain this variable, respondents were asked the question, “Have you ever been screened for cervical cancer?” The response options were: “No, I have never been screened,” “Yes, once,” “Yes, more than once,” and “Don't know/Not sure.” For this study, this dependent variable was re-coded as “screened yes” and “screened no”; those who answered “don't know” were not considered. We also included 17 variables as covariates in this study. These variables were grouped into sociodemographic variables (women's age, marital status, level of education, parity, and place of residence) and variables related to women's autonomy, namely knowledge or awareness (knowledge of cervical cancer, cervical cancer screening, human papillomavirus (HPV), and the HPV vaccine), decision-making power (role of women, final say on all household decisions, authorization to undergo screening, decisions on major household and family purchases, decisions concerning healthcare and cervical cancer screening), and financial considerations (access to health centers, access to referral services in the capital, cost of testing for women).

### 3.4 Data analysis

Data were collected through a household survey. A total of 360 women aged 25–69 years were recruited and interviewed at baseline. For the purpose of the present analysis, only the 312 women who provided a definitive response (“Yes” or “No”) to the question “Have you ever had cervical cancer screening?” were included. Participants with missing or indeterminate responses were excluded from the analysis of screening uptake. Data analysis was performed using R software version 4.2.1. Qualitative variables were described as proportions with absolute and relative frequencies, while quantitative variables were described using the median and mean with standard deviation. Bivariate analysis was performed using statistical tests to compare frequencies. Variables with a *p*-value <0.25 were included in the multivariate logistic regression to assess the effect of independent variables on the outcome of interest, controlling for the effect of covariates. The Hosmer–Lemeshow test was used to verify the quality of the model. A *p*-value <0.05 was used to determine the presence of a statistically significant association with the dependent variable. Adjusted odds ratios (AORs) and their corresponding 95% confidence intervals (95% CIs) were calculated to measure the strength of association between independent variables and cervical cancer screening uptake.

## 4 Results

### 4.1 Characteristics

Of the 312 women, 78.3% had never been screened. The women's ages ranged from 25 to 69, with a mean age of 37.8 ± 10.3. Married women were in the majority at 93.3%. The results showed that 34.3% of women had five or more children. The number of women living in Dakar (56.7%) was higher than those living in Kédougou (43.3%). Among the women included in the study, 81.7% reported having heard of cervical cancer. However, awareness of cervical cancer does not necessarily imply knowledge of screening services, as 28.2% reported that they had never heard of a cervical cancer screening test. In addition, 67.9% of women had never heard of human papillomavirus (HPV), and 56.1% were unaware of the existence of an HPV vaccine (Table 2).

**Table 2:** Results of descriptive analysis.

Variables		Absolute frequencies (*n*)	Relative frequencies (%)
Marital status	Single	6	1.9
	Married (monogamous)	214	68.6
	Married (polygamous)	77	24.7
	Divorced or separated	5	1.6
	Widowed	10	3.2
Parity	Nulliparous	13	4.3
	Between 1 and 4	186	61.4
	5 and above	104	34.3
Level of education	None	132	42.3
	Primary education	100	32.1
	Secondary level	64	20.5
	University	16	5.1
Place of residence	Dakar	177	56.7
	Kédougou	135	43.3
Have heard of cervical cancer	Yes	255	81.7
	No	57	18.3
Have you ever heard of a cervical cancer screening test?	Yes	224	71.8
	No	88	28.2
Have you ever heard of human papillomavirus (HPV)?	Yes	100	32.1
	No	212	67.9
Have you ever heard of the human papillomavirus (HPV) vaccine?	Yes	137	43.9
	No	175	56.1
A woman's most important role is to take care of the home and cook for the family.	Yes	235	75.3
	No	77	24.7
A man should have the final say on all decisions in the home.	Yes	267	85.6
	No	45	14.4
A family member would not allow me to have cervical cancer screening	Yes	42	17.4
	No	199	82.6
Decisions concerning major purchases for the home and family	Myself	48	15.4
	Joint decision	77	24.7
	Someone else	187	59.9
Decisions about the healthcare you receive	Myself	62	19.9
	Joint decision	62	19.9
	Someone else	188	60.3
Decision regarding cervical cancer screening	Myself	159	51.0
	Joint decision	89	28.5
	My partner	64	20.5
Sometimes I avoid going to the health center because I cannot afford it	Yes	202	64.7
	No	110	35.3
If I were referred for specialized care in Dakar, I would not be able to go.	Yes	182	58.3
	No	130	41.7
Cervical cancer screening was expensive.	Yes	8	11.6
	No	61	88.4
Have already undergone screening for cervical cancer	Yes	67	21.7
	No	242	78.3

During the study, we found that 75.3% of women considered that a woman's most important role was to take care of the home and cook for the family, while 85.6% of women believed that a man should have the final say on all decisions in the home. 17.4% of these women said that a family member would not allow them to be screened for cervical cancer. In addition, the decision to undergo cervical cancer screening was made by the woman's partner in 20.5% of cases.

Among the women included in the study, 64.7% avoided going to the health facility for financial reasons. Also, 58.3% of women said that if they were referred for specialized care in Dakar, they would not be able to go, and 11.6% felt that cervical cancer screening was expensive.

### 4.2 Bivariate analysis

Our study found a statistically significant link between place of residence and cervical cancer screening (*p* < 0.001). The majority of women who had undergone cervical cancer screening had a secondary education level (35.9%), followed by those with a primary education level (22.2%). The relationship between educational level and cervical cancer screening was statistically significant (*p* = 0.010). There was also a statistically significant link between awareness variables and cervical cancer screening (*p* < 0.001). Among women who considered that the most important role was to take care of the home and family, only 17.6% had undergone cervical cancer screening, compared with 34.2% of those who thought otherwise. The relationship between this variable and cervical cancer screening was statistically significant (*p* < 0.001). Among those who said that a man should have the final say on all decisions, only 18.9% had undergone cervical cancer screening, compared to 37.8% of those who disagreed. The relationship between this variable and cervical cancer screening was statistically significant (*p* = 0.008). The relationship between decision-making for cervical cancer screening and cervical cancer screening uptake was statistically significant (*p* < 0.001) (Table 3).

**Table 3:** Results of the bivariate analysis.

		Cervical cancer screening				
Variable		Yes		No		*P* value
		Number	%	Number	%	
Place of residence	Dakar	52	29.4	125	70.6	<0.001
	Kédougou	15	11.4	117	88.6	
Level of education	None	19	14.6	111	85.4	0.010
	Primary level	22	22.2	77	77.8	
	Secondary level	23	35.9	41	64.1	
	University	3	18.8	13	81.2	
Parity	5 and above	23	22.5	79	77.5	0.969
	Between 1 and 4	40	21.6	145	78.4	
	Nulliparous	3	23.1	10	76.9	
Marital status	Single	1	16.7	5	83.3	0.460
	Divorced or separated	1	20.0	4	80.0	
	Married (monogamous)	45	21.1	168	78.9	
	Married (polygamous)	20	26.3	56	73.7	
	Widowed	0	0	9	100	
Awareness of CCU	No	1	1.7	56	98.2	<0.001
	Yes	66	26.2	186	73.8	
Awareness of a CCU screening test	No	2	2.3	85	97.7	< 0.001
	Yes	65	29.3	157	70.7	
Awareness of HPV?	No	34	16.3	175	83.7	<0.001
	Yes	33	33.0	67	67.0	
Awareness of the HPV vaccine	No	21	12.2	151	87.8	<0.001
	Yes	46	33.6	91	66.4	
Your most important role is taking care of the home and your family	No	26	34.2	50	65.8	0.004
	Yes	41	17.6	192	82.4	
A man should have the final say on all decisions	No	17	37.8	28	62.2	0.008
	Yes	50	18.9	214	81.1	
Decisions concerning major purchases for the home and family	Joint decision	22	28.9	54	71.1	0.208
	Someone else	36	19.5	149	80.5	
	Yourself	9	18.8	39	81.2	
Decisions regarding healthcare	Joint decision	18	29.5	43	70.5	0.139
	Someone else	34	18.3	152	81.7	
	Yourself	15	24.2	47	75.8	
Decision regarding cervical cancer screening	Someone else	3	4.84	59	95.2	<0.001
	My own decision	43	27.0	116	73	
	A split decision	21	23.9	67	76.1	
A family member would not allow me to have cervical cancer screening	No	5	2.51	194	97.5	0.590
	Yes	0	0	42	100	
Cervical cancer screening was expensive.	No	59	96.7	2	3.28	0.063
	Yes	6	75	2	25	
Sometimes I avoid going to the health center because I cannot afford it	No	30	27.3	80	72.7	0.103
	Yes	37	18.6	162	81.4	
If I were referred for specialized care in Dakar, I would not be able to go.	No	31	23.8	99	76.2	0.518
	Yes	36	20.1	143	79.9	

### 4.3 Multivariate analysis

Compared with women whose partners made the decision regarding cervical cancer screening, women who reported making the decision jointly with their partner were significantly more likely to have undergone screening (AOR: 5.34; 95% CI: 1.35, 21.21). Similarly, women who reported making the decision themselves had higher odds of having undergone screening (AOR: 10.07; 95% CI: 2.72, 37.33). Women who were aware of the existence of a cervical cancer screening test were also more likely to have undergone screening than those who were not aware of it (AOR: 7.67; 95% CI: 1.41, 41.61). In contrast, women residing in Dakar were more likely to have undergone cervical cancer screening than those residing in the Kédougou region (AOR: 0.40; 95% CI: 0.17, 0.96) (Table 4).

**Table 4:** Results of multivariate analysis.

Variable	*P* value	OR crude	CI at 95%	*P* value	Adjusted OR	CI at 95%
Age	<0.001	1.04	(1.02, 1.07)	0.053	1.03	(1, 1.07)
Place of residence						
Kédougou	–	–	–	–	–	–
Dakar	<0.001	0.31	(0.16, 0.58)	0.038	0.40	(0.17, 0.96)
Level of education						
None	–	–	–	–	–	–
Primary level	0.139	1.67	(0.85, 3.29)	0.034	1.72	(0.79, 3.76)
Secondary level	<0.001	3.28	(1.62, 6.63)	–	3.21	(1.3, 7.95)
University	0.664	1.35	(0.35, 5.18)	–	0.72	(0.16, 3.33)
Awareness of CCU						
No	–	–	–	–	–	–
Yes	0.003	19.87	(2.7, 146.38)	0.695	1.58	(0.15, 16.89)
Awareness of a CCU screening test						
No	–	–	–	–	–	–
Yes	<0.001	17.60	(4.2, 73.64)	0.004	7.67	(1.41, 41.61)
HPV awareness						
No	–	–	–	–	–	–
Yes	0.001	2.54	(1.45, 4.42)	0.634	1.22	(0.54, 2.73)
Awareness of the HPV vaccine						
No	–	–	–	–	–	–
Yes	<0.001	3.63	(2.04, 6.48)	0.137	1.86	(0.82, 4.26)
Your most important role is to take care of the home and your family						
No	–	–	–	–	–	–
Yes	0.003	0.41	(0.23, 0.73)	0.519	0.75	(0.32, 1.79)
A man should have the final say on all decisions						
No	–	–	–	–	–	–
Yes	0.006	0.38	(0.2, 0.76)	0.737	0.84	(0.32, 2.26)
Decision regarding CCU screening						
Partner decision	–	–	–	–	–	–
My decision	0.001	7.29	(2.17, 24.49)	<0.001	10.07	(2.72, 37.33)
Shared decision split	0.005	6.16	(1.75, 21.71)	–	5.34	(1.35, 21.21)

## 5 Discussion

Overall, the prevalence of cervical cancer screening in our population remains low (21.7%). This level is comparable to that reported in a study conducted in five sub-Saharan African countries (19.0%) (6). However, it is higher than the prevalence observed in another study examining women's healthcare decision-making and cervical cancer screening uptake in the same region (13.4%) (12). These differences may be explained by variations in study settings, population characteristics, and access to cervical cancer screening services across countries and regions.

The findings of our study highlight the significant influence of women's decision-making power on the uptake of cervical cancer screening. Women who actively participate in decisions regarding their health are more likely to undergo screening than those whose healthcare decisions are made by their partner or another individual. In this context, it is consistent to observe that the Dakar region, where a higher proportion of women are involved in healthcare decision-making, also shows a higher prevalence of screening compared to the Kédougou region.

These findings can be interpreted in light of Connell's theory of gender and power, which suggests that male dominance particularly through control over decision-making and economic resources within the household constitutes a structural barrier to women's autonomy and access to maternal health services. In the study by Koné-Péfoyo and Rivard, this power dynamic limits women's ability to decide when and under what conditions to seek care, thereby contributing to the underutilization of maternal health services (13).

Our results are also consistent with those of Idris et al., who, in a recent systematic review, showed that women with greater decision-making autonomy particularly those who make decisions about their own health are more likely to seek and use healthcare services (14).

A possible explanation for this association is that women whose healthcare decisions are dominated by their partners may face greater resistance to screening, particularly due to limited understanding of cervical cancer and the importance of screening as a secondary preventive measure (15). Furthermore, in African settings, sociocultural norms and gender roles strongly influence women’s decision-making and access to healthcare services, especially in the context of reproductive health (16).

Our results also indicate that having heard about cervical cancer screening is associated with a higher likelihood of undergoing screening. This finding is consistent with previous studies, which have demonstrated the important role of knowledge in women's decision to be screened (17, 18). In our study, the majority of participants reported having heard about screening, which is a crucial factor, as greater awareness of preventive strategies promotes the adoption of appropriate health behaviors and facilitates adherence to health recommendations (19). However, other studies have shown that knowledge about cervical cancer remains limited in the general population, particularly regarding its link to sexually transmitted infections and the availability of screening services at the community level (19). This suggests that strengthening national awareness campaigns could contribute to improving health-seeking behaviors. In addition, our study shows that women living in the Kédougou region are the least likely to undergo cervical cancer screening. This finding is consistent with the work of Kangmennaang et al., who reported that women residing in rural areas are less likely to be screened compared to those living in urban areas (20). Since screening services are generally more accessible in urban settings, women in rural areas are at a clear disadvantage (21). Furthermore, several studies have highlighted that rural areas often lack adequate transportation infrastructure, which constitutes an additional barrier to accessing screening services (6). It is also important to note that women living in rural areas typically have limited access to information regarding the benefits, procedures, and availability of cervical cancer screening services (6, 22). As a result, these women often have insufficient knowledge of the disease and may perceive little value in screening, particularly in the absence of symptoms (23).

### 5.1 Limitations

This study has several limitations. The analysis relies primarily on self-reported data from participants and does not explicitly incorporate the perspectives of male partners, although reproductive health decisions may, depending on household dynamics and sociocultural norms, involve negotiation within couples. The use of self-reported data may be subject to recall bias or social desirability bias. To reduce the likelihood of such biases, standardized data collection tools and interviewer training were used; however, these measures could not eliminate bias entirely. In addition, some estimates were associated with relatively wide confidence intervals, suggesting a degree of imprecision that may be related to the limited number of screened women in certain categories. These findings should therefore be interpreted with caution. Furthermore, the study was conducted in two regions with contrasting urban and rural contexts, which may limit the generalizability of the findings to the entire country. Nevertheless, these limitations do not undermine the results but rather highlight important directions for future research, particularly the need to further integrate male involvement and couple-level decision-making into cervical cancer prevention strategies.

## 6 Conclusion

In conclusion, cervical cancer screening uptake remains low in both Dakar and Kédougou, highlighting persistent gaps in the use of preventive health services among women in Senegal. The findings of this study show that women who are involved in decision-making processes regarding their health are significantly more likely to undergo screening. This association underscores the importance of women's autonomy and agency in shaping health-seeking behaviors. In this context, public health interventions should prioritize approaches that strengthen women's capacity to make autonomous or shared health decisions. Particular attention should also be given to vulnerable groups, especially women with low levels of education and those living in rural areas, who are less likely to access screening services. In addition, improving women's knowledge of cervical cancer represents a key lever that can be used to strengthen awareness efforts and increase screening uptake. By addressing both individual determinants and structural factors influencing healthcare-seeking behaviors, Senegal can move toward more equitable and broader participation in cervical cancer prevention and control strategies.

## Data Availability

The raw data supporting the conclusions of this article will be made available by the authors, without undue reservation.
